# First Experimental *In Vivo* Model of Enhanced Dengue Disease Severity through Maternally Acquired Heterotypic Dengue Antibodies

**DOI:** 10.1371/journal.ppat.1004031

**Published:** 2014-04-03

**Authors:** Jowin Kai Wei Ng, Summer Lixin Zhang, Hwee Cheng Tan, Benedict Yan, Julia Maria Martinez Gomez, Wei Yu Tan, Jian Hang Lam, Grace Kai Xin Tan, Eng Eong Ooi, Sylvie Alonso

**Affiliations:** 1 Department of Microbiology, Yong Loo Lin School of Medicine, Life Sciences Institute, National University of Singapore, Singapore; 2 Immunology Programme, Yong Loo Lin School of Medicine, Life Sciences Institute, National University of Singapore, Singapore; 3 Defence Science Organization National Laboratories, Singapore; 4 Progamme in Emerging Infectious Diseases, Duke-NUS Graduate Medical School, Singapore; 5 Department of Pathology, National University Health System and National University of Singapore, Singapore; Washington University School of Medicine, United States of America

## Abstract

Dengue (DEN) represents the most serious arthropod-borne viral disease. DEN clinical manifestations range from mild febrile illness to life-threatening hemorrhage and vascular leakage. Early epidemiological observations reported that infants born to DEN-immune mothers were at greater risk to develop the severe forms of the disease upon infection with any serotype of dengue virus (DENV). From these observations emerged the hypothesis of antibody-dependent enhancement (ADE) of disease severity, whereby maternally acquired anti-DENV antibodies cross-react but fail to neutralize DENV particles, resulting in higher viremia that correlates with increased disease severity. Although *in vitro* and *in vivo* experimental set ups have indirectly supported the ADE hypothesis, direct experimental evidence has been missing. Furthermore, a recent epidemiological study has challenged the influence of maternal antibodies in disease outcome. Here we have developed a mouse model of ADE where DENV2 infection of young mice born to DENV1-immune mothers led to earlier death which correlated with higher viremia and increased vascular leakage compared to DENV2-infected mice born to dengue naïve mothers. In this ADE model we demonstrated the role of TNF-α in DEN-induced vascular leakage. Furthermore, upon infection with an attenuated DENV2 mutant strain, mice born to DENV1-immune mothers developed lethal disease accompanied by vascular leakage whereas infected mice born to dengue naïve mothers did no display any clinical manifestation. *In vitro* ELISA and ADE assays confirmed the cross-reactive and enhancing properties towards DENV2 of the serum from mice born to DENV1-immune mothers. Lastly, age-dependent susceptibility to disease enhancement was observed in mice born to DENV1-immune mothers, thus reproducing epidemiological observations.

Overall, this work provides direct *in vivo* demonstration of the role of maternally acquired heterotypic dengue antibodies in the enhancement of dengue disease severity and offers a unique opportunity to further decipher the mechanisms involved.

## Author Summary

Dengue (DEN) is an arthropod-transmitted viral disease which affects approximately 390 million individuals in the tropical and subtropical world annually. DEN clinical manifestations range from mild febrile illness (dengue fever) to life-threatening dengue hemorrhagic/dengue shock syndrome (DHF/DSS). Epidemiological observations indicate that infants born to dengue immune mothers are at greater risk to develop the severe form of the disease (DHF/DSS) upon infection with any serotype of dengue virus (DENV). It was proposed that the presence of maternally acquired DENV specific antibodies cross react but fail to neutralize DENV particles, resulting in higher viremia that correlates with increased disease severity. Direct experimental evidence supporting this antibody-dependent enhancement (ADE) hypothesis has been missing. Furthermore, a recent epidemiological report has challenged the influence of maternally acquired antibodies in disease outcome. Here, we have developed a mouse model of ADE where DENV2-infected mice born to DENV1 immune mothers displayed enhanced disease severity compared to DENV2-infected mice born to dengue naïve mothers. This is a long-overdue direct experimental evidence of the role of maternally acquired antibodies in dengue disease outcome. It provides a unique opportunity to dissect the mechanisms involved in this phenomenon.

## Introduction

Dengue (DEN) is the most prevalent arthropod-borne viral infection in the world [1]. Approximately 3 billion people who are living in the tropical and subtropical regions from Southeast Asia, the Pacific and the Americas are at risk of infection [1]–[3]. A recent meta-analysis using cartographic approaches estimates 390 million dengue infections per year including 96 million with clinical manifestations [4]. This number is more than three times higher than the previous dengue burden estimated by the World Health Organization [5]. With no licensed drug or vaccine, DEN represents a serious public health concern and economic burden for societies.

The etiological agent of DEN, dengue virus (DENV), belongs to the genus Flavivirus within the *Flaviviridae* family, which also includes Japanese encephalitis virus (JEV), West Nile virus (WNV), and yellow fever virus. DENV is an enveloped virus with a single-stranded, positive-sense 10.7 kb RNA genome. It is translated as a single polyprotein that is cleaved by viral and host proteases into three structural proteins (capsid [C], pre-membrane/membrane [prM/M] and envelope [E], and seven non-structural proteins (NS1, NS2A, NS2B, NS3, NS4A, NS4B and NS5) [6]. There are four antigenically distinct serotypes of DENV (DENV1-4) that may co-circulate in the same geographical area [1], [3]. The virus is primarily transmitted to humans by the highly urbanised *Aedes aegypti* female mosquito which has spread globally due to increased trade and travel [7].

Human infection with one of the four DENV serotypes can result in either asymptomatic or symptomatic disease; the latter presents itself in a wide spectrum of clinical manifestations, ranging from mild acute febrile illness to self-limiting classical dengue fever (DF) to the severe dengue hemorrhagic fever/dengue shock syndrome (DHF/DSS) [8], [9]. The hallmark of DHF/DSS is the increased vascular permeability that results in fluid loss which may progress to hypovolemic shock. Clinical management of DHF/DSS patients consists of isotonic fluid resuscitation and blood/platelet transfusion when appropriate [8], [9]. Specific to DEN, viremia is transient (around 7–10 days) and development of the severe forms of the disease typically occurs during defervescence when the virus is almost cleared from the blood circulation [2].

Despite increasing interest from the scientific community worldwide, the mechanisms involved in DEN pathogenesis, and in particular DHF/DSS, remain unclear with increasing contradictory and controversial findings [10]. This is partly due to the lack of a robust animal model of DEN which recapitulates the clinical manifestations and disease kinetic as seen in dengue patients [11]. Thus currently, most of the knowledge on the mechanisms involved in dengue pathogenesis has been derived from both *in vitro* systems and epidemiological observations although a number of mechanistic hypotheses could be experimentally confirmed *in vivo* in mouse models [11].

Whereas infection with one DENV serotype confers an individual life-long protection against that particular DENV serotype, it does not cross-protect against the other DENV serotypes [2], [7]. Instead, epidemiological studies conducted worldwide over five decades have indicated that the vast majority of DHF/DSS cases occur upon secondary infection with a heterologous DENV serotype [12]–[15]. Mechanistically, these epidemiological observations could be explained by the antibody-dependent enhancement (ADE) of infection hypothesis, whereby actively (upon primary infection) or passively (through maternal transfer) acquired anti-DENV antibodies cross react but fail to neutralize heterotypic DENV particles [12]. Furthermore, it was also proposed that sub-neutralizing concentrations of homotypic DENV antibodies also trigger ADE [12]. Mechanistically, antibody-opsonized DENV gains entry into Fc receptor (FcR)-bearing cells such as monocytes resulting in increased viral replication which in turn triggers the massive release of inflammatory and vasoactive mediators that contribute to the disease severity [16]–[18]. *In vitro* studies using heterotypic or sub-neutralizing concentrations of homotypic immune sera or monoclonal antibodies and FcR-bearing cells have supported the ADE hypothesis, with higher virus production and higher levels of cytokines such as TNF-α or IL-1β compared to viral infection in absence of the antibodies [19]–[22]. *In vivo*, passive transfer of a monoclonal cross-reactive anti-DENV antibody in rhesus monkeys infected with DENV4 resulted in significantly increased viremia titers [23]. In an acute fatal mouse model of severe DEN, passive administration of heterotypic anti-DENV antibodies to DENV2-infected mice led to increased virus titers, cytokine storm, lower platelet counts, increased vascular permeability, intestinal hemorrhage and reduced survival rate, compared to the infected untreated control animals [24], [25].

Perhaps the strongest clinical evidence for a role for antibodies in DEN pathogenesis comes from the observation of increased risk of DHF/DSS in infants at 5–9 months of age where maternal antibodies against DENV wane to sub-neutralizing levels [26]–[30]. While infection with a heterologous DENV serotype in young children or adults would trigger anamnestic but cross-reactive responses from both B and T cells [29], [30], only maternal antibodies but not T cells cross the placenta to the infant. This indicates that disease enhancement cannot be due to other factors but sub-neutralizing levels of DENV antibodies in this case [31]. However, a more recent epidemiological study has challenged this hypothesis whereby no significant association was found between DENV3 ADE activity at illness onset and the development of DHF in infants compared with less severe symptomatic illness [32]. Thus, there is an urgent need to address this controversy experimentally as the ADE hypothesis is the most widely proposed explanation for clinical and epidemiological observations. This work describes a unique animal model of enhanced disease severity upon primary DENV infection of mice born to mothers immune to a heterotypic DENV strain.

## Materials and Methods

### Ethics statement

All the animal experiments were carried out under the guidelines of the National Advisory Committee for Laboratory Animal Research (NACLAR) in the AAALAC-accredited NUS animal facilities (http://nus.edu.sg/iacuc/). NUS has obtained a license (#VR008) from the governing body Agri-Food & Veterinary Authority of Singapore (AVA) to operate an Animal Research Facility. The animal experiments described in this work were approved by the IACUC from National University of Singapore under protocol number 009/09. Non-terminal procedures were performed under anesthesia, and all efforts were made to minimize suffering of the animals.

### Virus strains and growth conditions

D2Y98P-PP1 is a DENV2 strain derived from a 2000 Singapore clinical isolate (Genbank accession number #JF327392) [33]. DENV1 [Dengue 1 05K3903DK1 (Genbank accession number #EU081242)] was isolated from a patient during a DEN outbreak in Singapore in 2005 [34]. MT5 strain was derived from D2Y98P-PP1 virus through a single amino acid substitution (Phe->Leu) at position 52 of NS4B protein [33]. All the DENV strains were propagated in the *Aedes albopictus* cell line C6/36 (American Type Culture Collection [ATCC #CRL-1660]). C6/36 cells were maintained in Leibovitz's L-15 medium (GIBCO) supplemented with 5% fetal calf serum (FCS), and virus propagation and harvest were carried out as described previously [35]. Virus stocks were stored at −80°C. Virus titres of these virus stocks were determined by plaque assay in BHK-21 cells as described below.

UV-inactivation of DENV particles was performed using a handheld 6 watts shortwave UV lamp (product no. UVG-54) for 10 mins at a distance of 15 cm inside a biological safety cabinet. Plaque assay was performed to ensure that the DENV particles were inactivated.

### Plaque assay

Plaque assay was carried out in BHK-21 cells as described previously [35]. Briefly, 2×10^5^ cells BHK-21 were seeded in 24-well plates (NUNC, NY, USA). BHK-21 monolayers were infected with 10-fold serially diluted viral suspensions ranging from 10^−1^ to 10^−8^. After 1 h incubation at 37°C and 5% CO_2_, the medium was decanted and 1% (w/v) carboxymethyl cellulose was added to the wells. After 4 days (D2Y98P-PP1) or 5 days (DENV1) incubation at 37°C and 5% CO_2_, the cells were fixed with 4% paraformaldehyde and stained with 1% crystal violet. The plates were rinsed thoroughly, dried and the plaques were scored visually and expressed as the number of plaque forming units (PFU). Triplicate wells were run for each dilution of each sample. The limit of detection for the plaque assay was set at 10 PFU per ml.

### Determination of virus loads in plasma by real-time PCR

Blood samples were collected in 0.4% sodium citrate and centrifuged for 5 min at 6,000 g to obtain plasma. The presence of infectious viral particles was determined by real-time PCR as follows. DENV2 viral RNA was extracted from 100 ul of infected mouse serum using QIAviral RNA extraction kit (Qiagen), according to the manufacturer's protocol. Reverse transcription was then performed on 5 ul of total RNA using iScript cDNA synthesis kit (Bio-Rad Laboratories). Real-time PCR (RT-PCR) was performed on ABI Prism 7500 sequence detector (Applied Biosystem) over 40 cycles with an annealing temperature of 60°C using D2Y98P-PP1 NS4B primers as described previously [35]. Samples were run in triplicates. At least 2 independent experiments were conducted for the respective experiments. Data are expressed in Log_10_ PFU Equiv. DEN RNA/mL serum.

### Determination of virus loads in organs by real-time PCR

Virus loads in the organs from DENV-infected mice were determined by RT-PCR. Briefly, at Day 5 post-infection, mice were euthanized and perfused extensively with sterile PBS. Pooled left and right brachial and axillary lymph nodes, intestine, spleen, and liver were harvested and stored in RNAlater solution (Ambion). Within 24 hours, total RNA was extracted from 30 mg of the respective organs using Qiagen RNeasy kit, according to the manufacturer's protocol. Reverse transcription was performed on 1000 ng of total RNA using iScript cDNA synthesis kit (Bio-Rad Laboratories). RT-PCR amplification was conducted with ABI Prism 7500 sequence detector as described above. Samples were run in triplicates. 18s rRNA primers were also used as internal reference for data normalization. The following primers were used: NS4B forward primer (FP), 5′-AACCGAGATGGGTTTCCTGGAA-3′; NS4B reverse primer (RP), 5′-TTCAAACTTTGGATCATAGGGT-3′; 18srRNA (FP), 5′-CGGCTACCACATCCAAGGAA-3′; 18srRNA (RP), 5′-GCTGGAATTACCGCGGCT-3′. Data are expressed in Log_10_ PFU Equiv. DEN RNA/organ.

### ADE infection mouse model

AG129 [129/Sv mice deficient in both alpha/beta (IFN-α/β) and gamma (IFN-γ) interferon receptors] breeders were obtained from B&K Universal (UK). They were housed and bred under specific pathogen-free conditions in individual ventilated cages. Six-week old female AG129 mice were infected with 10^6^ PFU of DENV1 per mouse via the subcutaneous (sc) route (0.1 ml in sterile PBS) which led to asymptomatic infection. One week post-infection, after virus clearance, the females were allowed to mate with naïve 6-week old AG129 males and pups were weaned out 21 days later. Uninfected AG129 females were also used to give birth to naïve controls. At 2, 5 or 8 weeks of age (as indicated), mice born to DENV1 immune or naive mothers were administered with 10^3^ PFU of DENV2 (D2Y98P-PP1 or MT5 strain) via the sc route (0.1 ml in sterile PBS). The clinical symptoms were scored as follows: 0 - at healthy state, 1 - signs of ruffled fur, 2 - hunched back, 3 –severe diarrhea, 4 - moribund stage, 5 - severe weight loss. The infected animals were monitored daily (clinical score between 0–2) and every 12 hours (clinical score from 3 onwards). Survival rate was derived from the number of mice that were euthanized at moribund stage as evidenced by severe diarrhea, lethargy, and sharp body weight loss as described previously [35], [36].

### Histology

Mice were euthanized and tissues (intestines and liver) were harvested and immediately fixed in 10% formalin in PBS at the indicated time points. Fixed tissues were paraffin embedded and stained with Hematoxylin and Eosin (H&E).

### Hematology

Mouse blood samples were collected in K2EDTA and serum tubes (Greiner). Whole blood was immediately analyzed for cell counts using automated hematology analyzer Cell Dyn – 3700 (Abbott). Serum alanine (ALT) and aspartate (AST) aminotransferases levels were quantified using chemistry analyzer COBAS C111 (ROCHE).

### Assessment of vascular leakage

Vascular leakage was assessed using Evans Blue dye as a marker for albumin extravasation as described previously [35]. Briefly, 0.2 ml of Evans blue dye (0.5% w/v in PBS) (Sigma Aldrich) were injected intravenously into the anesthetized mice. After 2 h, the animals were euthanized and extensively perfused with PBS. The tissues were harvested and weighed prior to dye extraction using N,N-dimethylformamide (Sigma; 4 ml/g of wet tissue) at 37°C for 24 h after which absorbance was read at 620 nm. Data are expressed as fold change in OD620nm per gram of wet tissue compared to control group or as absolute absorbance values per gram of wet tissue.

### Detection of cytokines and other soluble mediators

TNF-α, Interleukin-6 (IL-6), VEGFA and MMP-9 levels were quantified in the mouse serum using commercially available detection kits (R&D systems) and according to the manufacturer's instructions.

### Production of DENV1 immune serum

Six-week old female AG129 mice (n = 10) were sc infected with 10^6^ PFU of DENV1 per mouse. Eight weeks post-infection, mice were euthanized and serum was collected by cardiac puncture. Serum was also obtained from age-matched uninfected control animals. Pooled sera from each group were heat-inactivated, titrated by ELISA and stored at −80°C until use.

### Passive transfer of DENV1 immune serum

Five-week old AG129 mice were ip administered with 100 µl/mouse of neat heat-inactivated DENV1 immune serum or naive serum. Mice were sc infected with 10^3^ PFU of D2Y98P-PP1 24 h post-administration.

### Anti-TNFα antibody treatment

Five-week old AG129 mice born to DENV1 immune mothers were sc infected with 10^3^ PFU of D2Y98P-PP1. At Day 1 and Day 2 post-infection, mice were injected intraperitoneally (ip) with 100 µg of anti-TNFα (eBioscience, Cat. no. 167322-85) or 100 µg Rat IgG1 K isotype control (eBioscience, Cat. no. 16-4301-85) per mouse as described previously [37].

### Measurement of DENV specific total IgG and IgG isotypes

The levels of systemic IgG antibodies against DENV1 or DENV2 were determined by enzyme-linked immunosorbent assay (ELISA). Briefly, 96-well plates (Corning costar, NY, USA) were coated overnight at 4°C with 10^5^ PFU of UV-inactivated DENV1 or DENV2 in 0.1M NaHCO3 buffer at pH 9.6. Diluted (1/50) serum samples were added to the wells and incubated for 1 h at 37°C. HRP-conjugated goat anti-mouse IgG (H+L) (Bio-rad) secondary antibody was used at a 1∶3,000 dilution. Detection was performed using O-phenylenediamine dihydrochloride substrate SigmaFast (Sigma Aldrich) according to the manufacturer's instructions. The reaction was stopped upon adding 75 µl of 1M H2SO4 and absorbance was read at 490 nm using an ELISA plate reader (Bio-rad model 680).

For IgG isotypes analysis, secondary goat HRP-conjugated anti-mouse IgG1, IgG2a, IgG2b and IgG3 (Jackson Immuno Research) were used at a 1∶3,000 dilution.

### *In vitro* ADE assay

Sera from 5-week old mice born to naïve or DENV1 immune mothers were inactivated at 56°C for 30 minutes. Sera diluted 1∶20 were incubated for 1 h at 37°C with DENV2 at a multiplicity of infection (MOI) of 10 before adding the suspensions to non-adherent human monocytes THP-1 cells in 96-wells flat bottom tissue culture plates (NUNC) as described previously [38]. At 72 h post infection, cells were harvested and clarified by centrifugation. The virus titer in culture supernatants was determined by plaque assay on BHK-21 cells.

### *In vitro* neutralizing assay and PRNT_50_

Serial dilutions (2-fold, starting 1/10 dilution) of heat-inactivated serum from mice born to DENV1 immune mothers was performed in a 96-well sterile flat bottom plate with RPMI 1640 with 2% FBS (life technologies). Each dilution (100 µl) was incubated at 37°C for 1 hour with 100 µl containing approximately 50 PFU of DENV2. A positive control mix with virus alone was included. Plaque assay was then carried out in BHK-21 cells as described above and in triplicates in 24-well plates. The percentage of neutralization was determined by comparing the number of plaques obtained with each serum dilution to that obtained with the positive control. Data were plotted in Graphpad Prism (version 5.0a) and PRNT_50_ was determined by nonlinear regression as the serum dilution factor for which 50% reduction in the number of plaques was obtained.

### Statistical analysis

The results were analyzed using the unpaired Student *t* test. Differences were considered significant (*) at *p* value <0.05.

## Results

### AG129 mice born to DENV1 immune mothers and infected with a DENV2 lethal strain displayed enhanced disease severity

**Figure 1:**
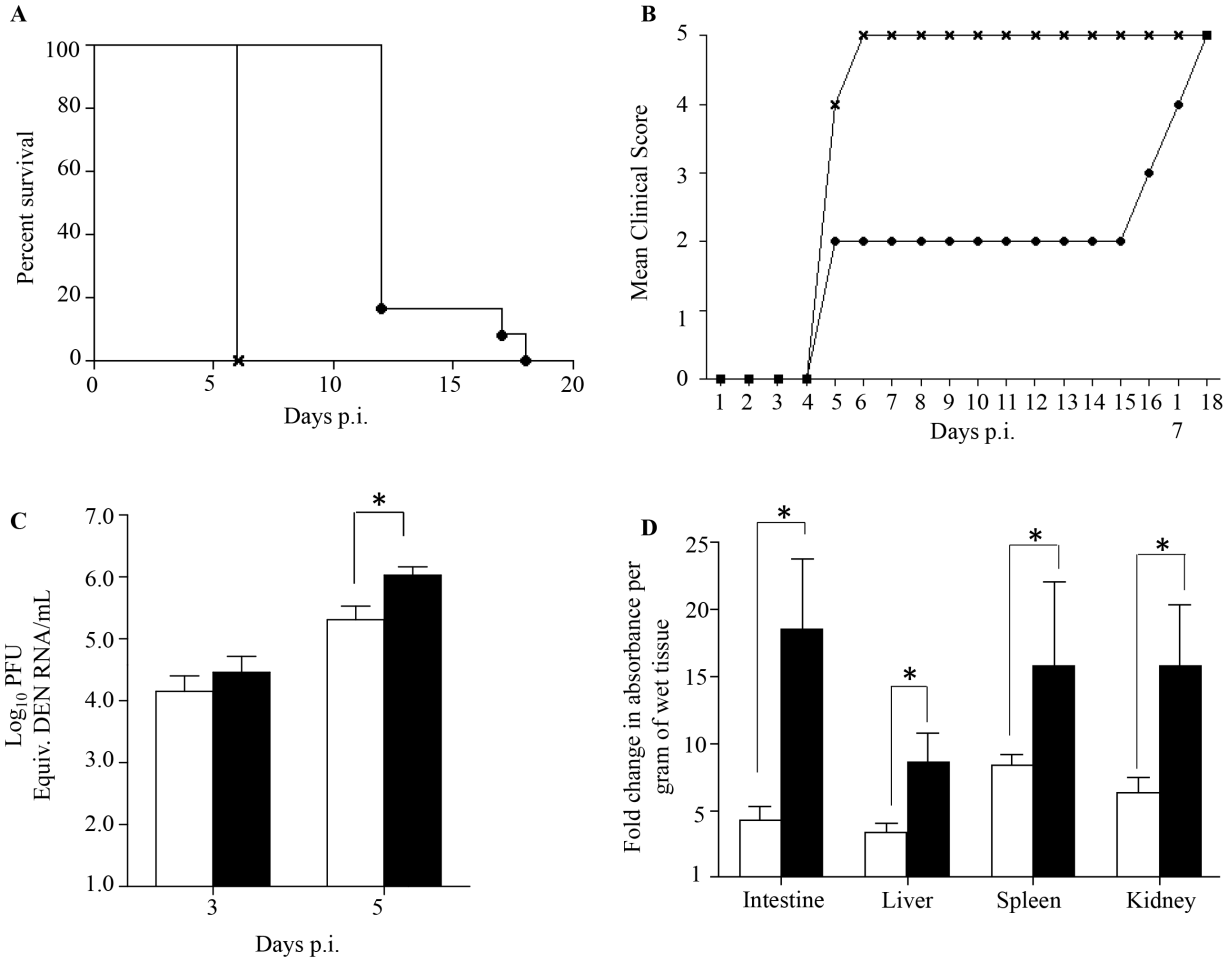
Survival rate, clinical score, viremia and vascular leakage in DENV2-infected mice born to either DENV1 immune or dengue naïve mothers. 5-weeks old AG129 mice born to either DENV1 immune or dengue naïve mothers were sc. infected with 10^3^ PFU of D2Y98P-PP1, a DENV2 strain. A) Survival Rate (n = 10 animals per group). B) Mean Clinical score. 0: Healthy; 1: Ruffled Fur; 2: Hunched back; 3: Severe Diarrhea; 4: Lethargic; 5: Moribund (n = 10 animals per group). C) Viremia. At Day 3 and 5 p.i., 5 mice per group per time point were bled by cardiac puncture and the number of infectious viral particles was determined by real-time PCR. D). Vascular leakage. At moribund stage, 5 mice per group were intravenously injected with Evans's blue dye and euthanized 2 hours later. After careful and extensive systemic perfusion with PBS, various organs were harvested and processed for Evan's blue dye extraction and quantification. Results are expressed as fold changes compared to uninfected control animals. Legend: DENV2-infected mice born to dengue naive mothers (black circle or white bar); DENV2-infected mice born to DENV1 immune mothers (cross or black bar). *, p<0.05.

Adult female AG129 mice were infected sc. with 10^5^ PFU of a clinical isolate of DENV1 which led to asymptomatic transient viremia (Suppl. Fig. S1). At 7 days post-infection (p.i.), once the virus has been cleared from the circulation the mice were mated with dengue naïve AG129 adult males. At 5 weeks of age, the mice born to the DENV1 immune mothers were sc. infected with 10^3^ PFU of D2Y98P-PP1, a DENV2 strain that triggers severe dengue associated with vascular leakage and death as reported by us previously [35], [36]. The survival rate indicated that DENV2-infected mice born to DENV1 immune mothers died much earlier (at day 6 p.i.) as compared to age-matched DENV2-infected mice born to dengue naive mothers (day 12 to 18 p.i.) (Fig. 1A). Furthermore, mice born to DENV1 immune mothers did not display the clinical symptoms progression that was typically seen for the first four days upon primary infection with D2Y98P-PP1 virus including ruffled fur, hunched back, and severe diarrhea (Fig. 1B) [36]. Instead DENV2-infected mice born to DENV1 immune mothers rapidly progressed into lethargy and eventually reached moribund stage at day 6 p.i. at which point the animals were euthanized. No signs of paralysis were observed in both DENV2-infected groups throughout the course of the experiment.

These observations indicate that DENV2-infected mice born to DENV1 immune mothers experienced enhanced disease severity, similar to observations made with dengue infected children born to dengue immune mothers [26]–[30].

### DENV2-infected mice born to DENV1 immune mothers displayed higher viremia and increased vascular leakage compared to DENV2-infected mice born to naïve mothers

Previous studies have reported a direct correlation between high viremia and disease severity [39], [40]. Viremia was thus determined by real-time PCR at day 3 and 5 p.i. in DENV2-infected mice born to DENV1 immune or naïve mothers. At day 3 p.i. comparable virus loads were measured in both DENV2-infected groups, whereas significantly higher virus loads were measured at day 5 p.i. in the DENV2-infected mice born to DENV1 immune mothers (Fig. 1C). Together, these results thus support that the presence of maternal DENV1 specific antibodies circulating in DENV2-infected mice led to an increased production of infectious virus particles.

Furthermore, increased vascular permeability is a hallmark of severe dengue in humans which is also observed in our DENV2 primary infection mouse model [35], [36]. Vascular permeability was thus measured in DENV2-infected mice born to DENV1 immune or naïve mothers at moribund stage using Evan's blue dye extrusion assay [35]. A significantly higher vascular leakage in the moribund mice born to DENV1 immune mothers was observed in all the organs tested compared to moribund mice born to naïve mothers (Fig. 1D).

Together, these results indicate that the enhanced disease severity observed in DENV2-infected mice born to DENV1 immune mothers correlate with higher viremia and greater vascular leakage compared to mice born to naïve mothers.

### DENV1 immune serum enhanced disease severity upon DENV2 infection

**Figure 2:**
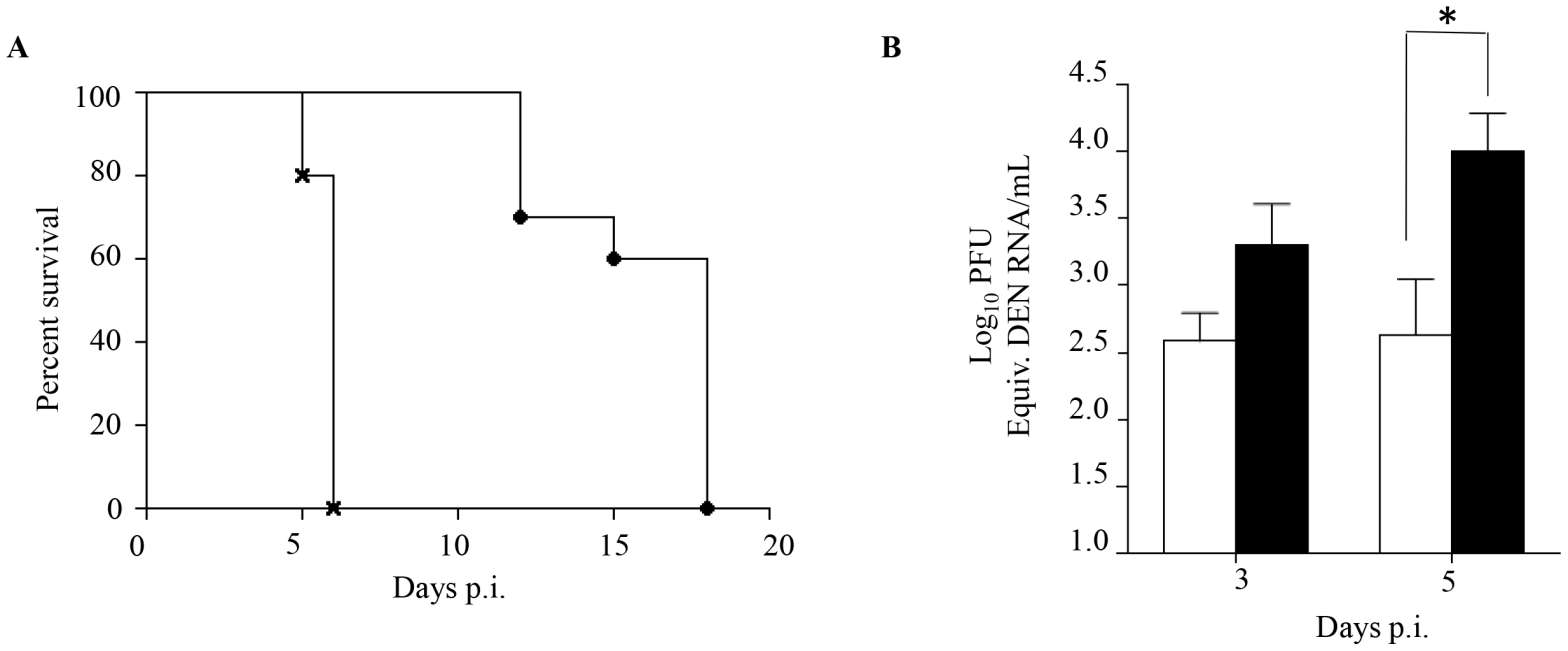
Passive transfer of DENV1 immune serum to naive AG129 mice prior to DENV2 infection. 5-weeks old naive AG129 mice born to naïve mothers were injected intraperitoneally with 100 µl of either naive or DENV1 immune serum obtained from naïve or DENV1-immune adult AG129 mice, respectively. One day later, the mice were sc. infected with 10^3^ PFU of D2Y98P-PP1. A) Survival rate (n = 10 mice per group). B) Viremia. At Day 3 and 5 p.i., 5 mice per group per time point were euthanized and blood was harvested by cardiac puncture. The virus titers in the serum were determined by real-time PCR. Legend: 5-weeks old AG129 mice passively transferred with naive immune serum (black circle or white bar); 5-weeks old AG129 mice passively transferred with DENV1 immune serum (cross or black bar). *, p<0.05.

Enhanced disease severity in DENV infected children born to dengue immune mothers has been proposed to be mediated by maternally transferred non neutralizing cross-reactive antibodies [12]. In order to confirm the role of maternal antibodies in our mouse model of dengue disease enhancement, a passive immune transfer experiment was carried out whereby naïve 5-week old AG129 mice were injected ip. with naïve or DENV1 immune serum one day prior to DENV2 infection. Similar to what was observed with DENV2-infected mice born to DENV1 immune mothers, DENV2-infected mice passively transferred with DENV1 immune serum died much earlier than DENV2-infected mice administered with naïve serum, with survival means of 6 and 18 days, respectively (Fig. 2A). Furthermore, significantly higher virus loads at day 5 p.i. were detected in mice that received DENV1 immune serum compared to the control animals (Fig. 2B), thus reproducing the viremia pattern seen with DENV2-infected mice born to DENV1 immune mothers (Fig. 1C).

Together, these results support that the enhanced dengue disease severity observed in DENV2-infected mice born to DENV1 immune mothers is caused by maternal antibodies that are circulating in these animals at the time of DENV2 infection.

### Histology, viral loads and blood parameters

**Figure 3:**
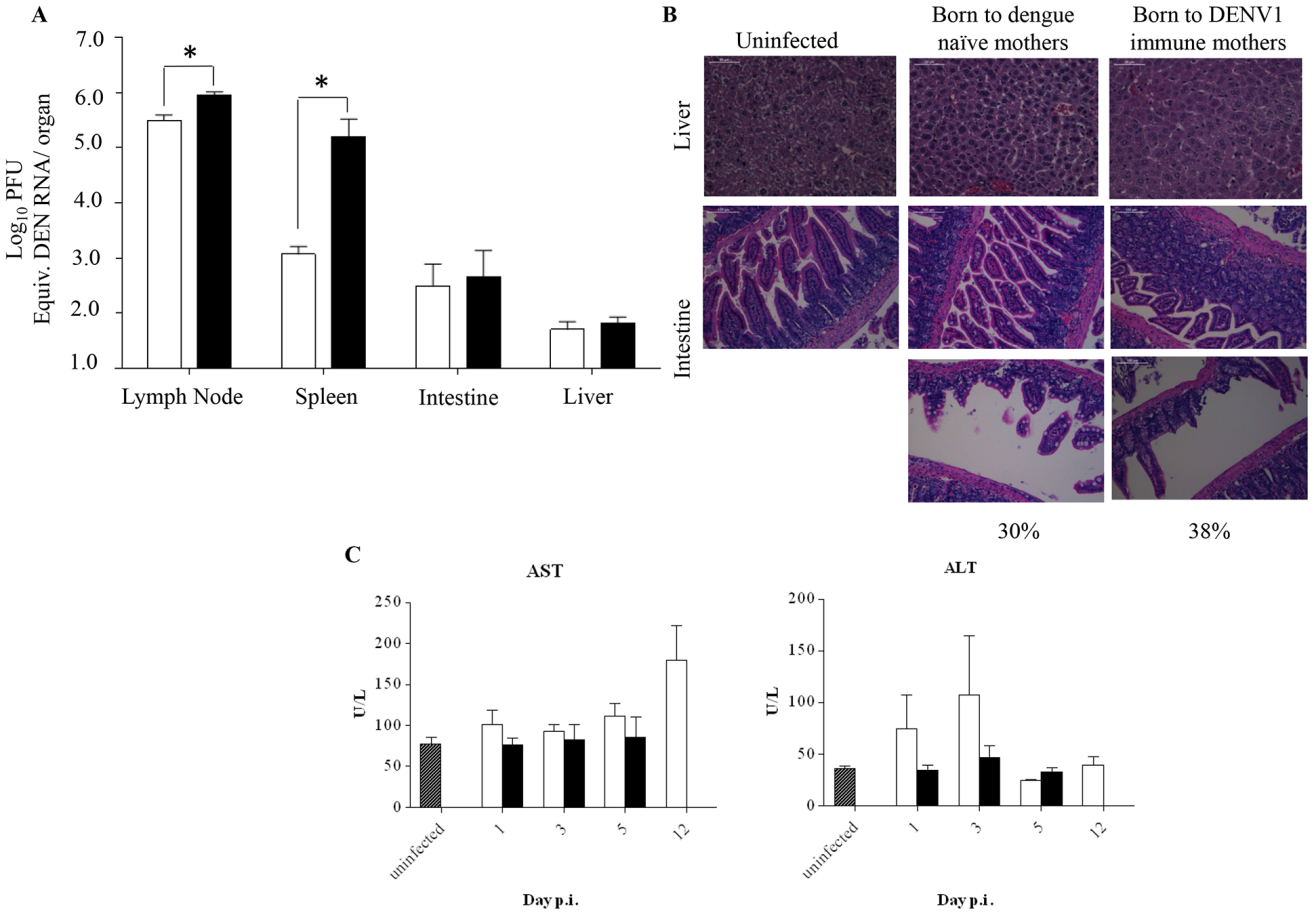
Viral load and histology analysis in DENV2-infected mice born to either DENV1 immune or dengue naïve mothers. 5-weeks old AG129 mice born to either DENV1 immune or dengue naive mothers were sc. infected with 10^3^ PFU of D2Y98P-PP1. A) Virus load in brachial/axillary lymph nodes, spleen, intestines and liver. At Day 5 p.i., 5 mice per group were euthanized and systemically perfused with PBS prior to organ harvest and processing for viral RNA extraction. The viral loads were measured by real time PCR. B) Histology analysis of the liver and intestines harvested at moribund stage, fixed and processed for H&E staining. Observations were made at 400× (liver) and 200× (intestine). Representative sections from 3 individual mice are shown. Three sections per organ were analyzed and 20 fields per section were viewed. C) Serum levels of aspartate (AST) and alanine (ALT) transaminases. At the indicated time points p.i., 5 mice per group per time point were euthanized and bled by cardiac puncture for AST and ALT quantification. Legend: DENV2-infected mice born to DENV1 immune mothers (black bar); DENV2-infected mice born to dengue naive mothers (white bar); uninfected age-matched mice born to dengue naïve mothers (hatched bar). *, p<0.05.

To further characterize the disease severity enhancement observed in DENV2-infected mice born to DENV1 immune mothers, we measured the viral load in various organs. Five days post-DENV2 infection, the animals were euthanized and perfused with PBS prior to organ harvest in order to avoid contamination by infectious DENV particles circulating in the blood that may artificially lead to over-estimation of the number of infectious particles detected in the organs, in particular in highly vascularized organs such as the spleen and liver. The results indicated that a significantly higher viral load was measured in the spleen but not in the lymph nodes, intestines and liver from DENV2-infected mice born to DENV1 immune mothers compared to DENV2-infected mice born to dengue naïve mothers (Fig. 3A). This data thus suggest that the antibody-mediated enhanced viral replication and output mainly occurs in the blood and spleen.

Furthermore, histological analysis showed no intestinal damage in DENV2-infected mice born to DENV1 immune or naïve mothers at moribund stage (Fig. 3B). However, signs of villi disintegration and detachment in some areas of the intestines were observed for 38% and 30% of the fields analyzed for each infected group respectively (Fig. 3B). Furthermore, no significant damage in the liver was observed for both DENV2-infected groups (Fig. 3B), with levels of alanine and aspartate transaminases not significantly different from the levels measured in the uninfected controls born to dengue naïve mothers (Fig. 3C).

**Figure 4:**
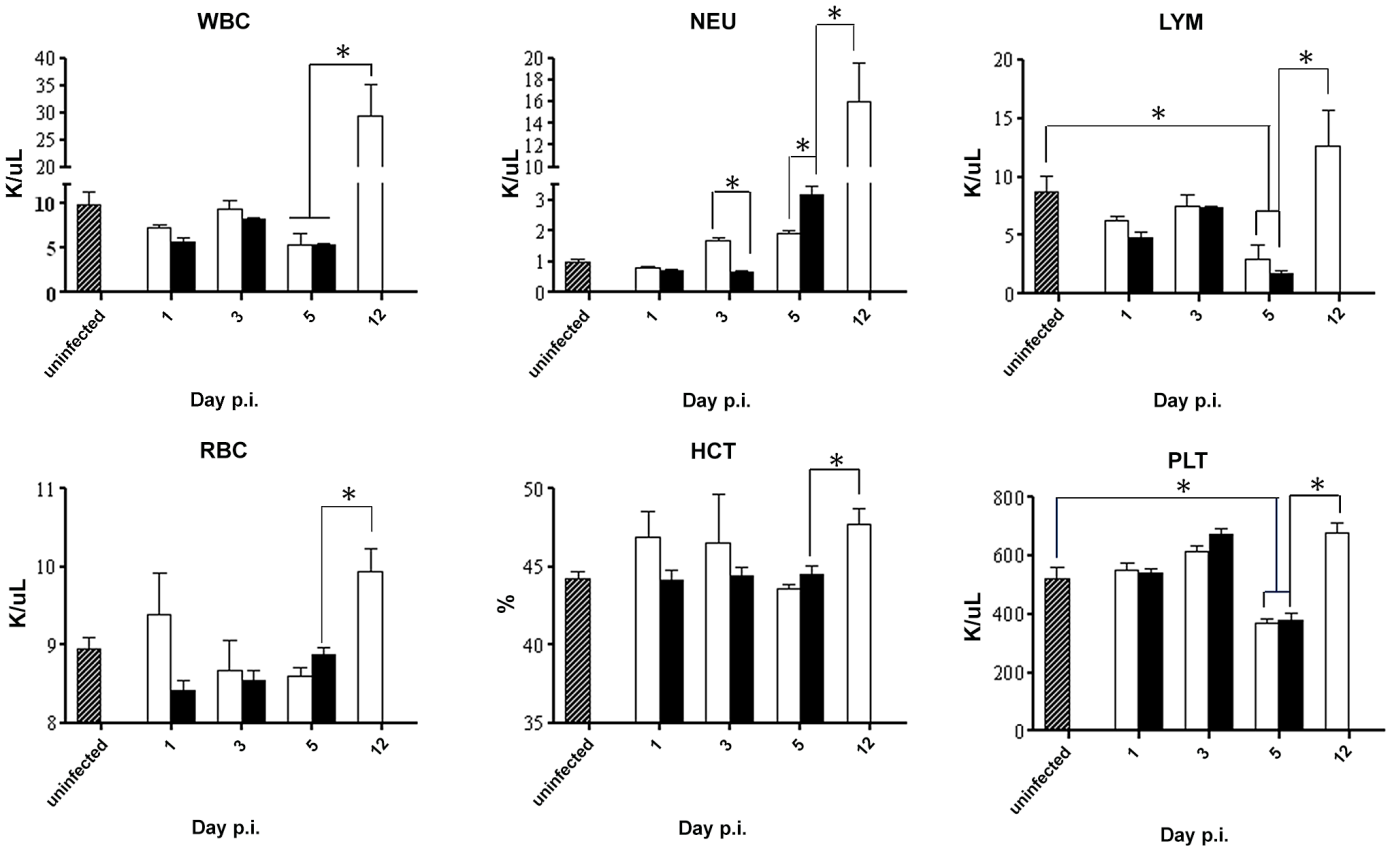
Blood parameters in DENV2-infected mice born to either DENV1 immune or dengue naïve mothers. 5-weeks old AG129 mice born to either DENV1 immune or naive mothers were sc. infected with 10^3^ PFU of D2Y98P-PP1. At the indicated time points p.i., 5 mice per group were euthanized and blood was harvested by cardiac puncture for measurement of various blood parameters including white blood cells (WBC), neutrophils (NEU), lymphocytes (LYM), red blood cells (RBC), hematocrit (HCT), and platelet (PLT) counts. Legend: DENV2-infected mice born to DENV1 immune mothers (black bar); DENV2-infected mice born to dengue naive mothers (white bar); uninfected age-matched mice born to dengue naïve mothers (hatched bar). *, p<0.05.

In addition, various blood parameters were assessed over time post-DENV2 infection. The levels of white blood cells (WBC), lymphocytes (LYM), red blood cells (RBC), hematocrit (HCT) and platelets (PLT) were comparable between both animal groups at day 1, 3, and 5 p.i. (Fig. 4). Notably, drops in lymphocyte (LYM) and platelets (PLT) concentrations were detected at day 5 p.i. which is consistent with previous reports in dengue patients [2]. A delayed increase in the neutrophil (NEU) concentration was observed in the group of mice born to DENV1 immune mothers compared to mice born to naïve mothers. Also, remarkably, when comparing both DENV2-infected groups at moribund stage, corresponding to day 5 p.i. for mice born to DENV1 immune mothers and day 12 p.i. for mice born to naïve mothers, the level of all the blood parameters monitored were found significantly higher in the latter group.

Together, these results indicate that enhancement of disease severity in this mouse model cannot be explained by aggravated tissue damage or profound changes in blood parameters. The hematological differences observed between DENV2-infected mice born to DENV1 immune versus naïve mothers is likely due to the differential disease progression rate between both animal groups whereby DENV2-infected animals born to DENV1 immune mothers die much more rapidly than the mice born to naïve mothers which therefore does not allow observing in the former group further increase in concentration of some of the blood parameters.

### DENV2-infected mice born to DENV1 immune mothers displayed higher levels of IL-6 and TNF-α than mice born to naïve mothers

**Figure 5:**
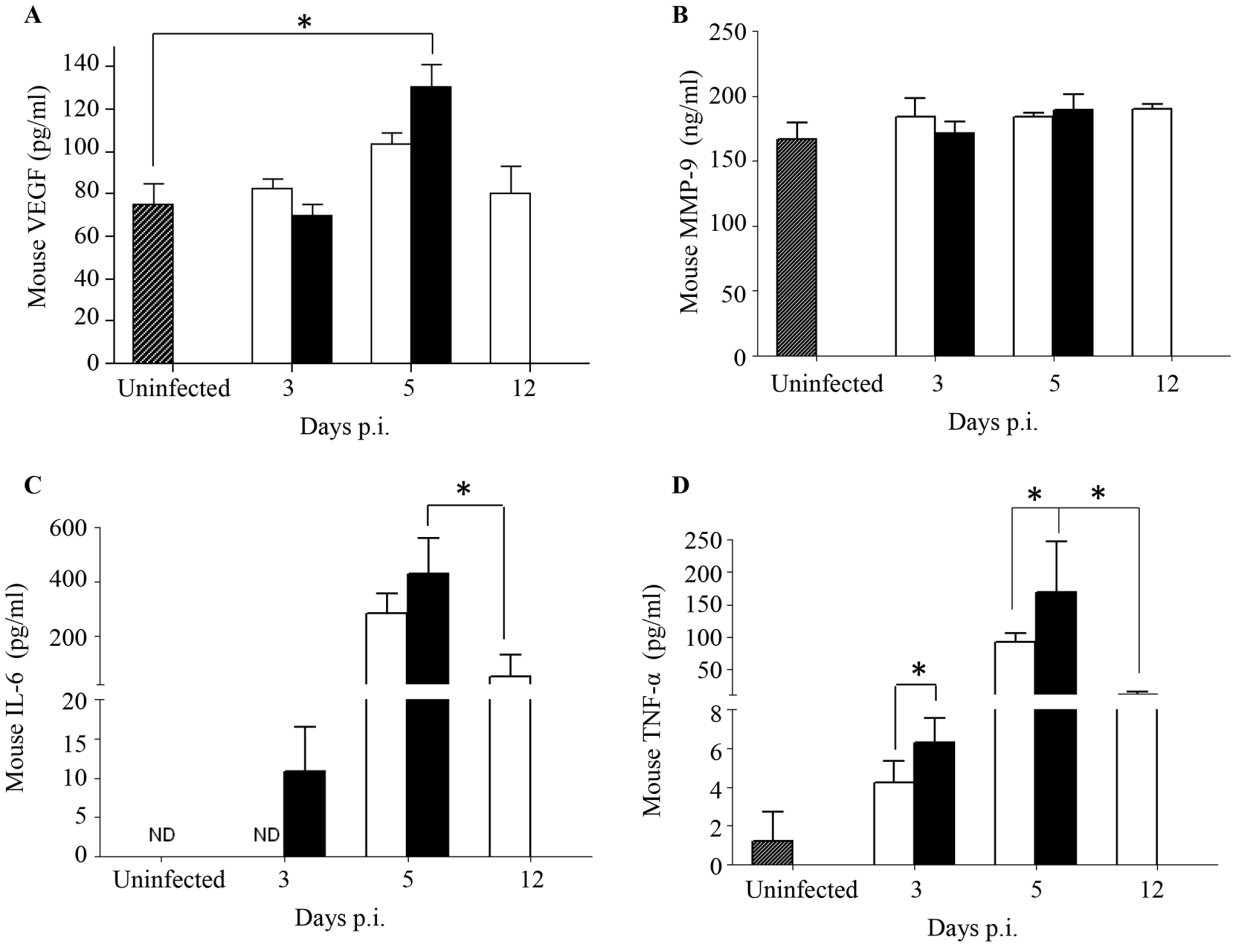
Systemic levels of soluble mediators in DENV2-infected mice born to either DENV1 immune or naïve mothers. 5-weeks old AG129 mice born to either DENV1 immune or naive mothers were infected sc. with 10^3^ PFU of D2Y98P-PP1. At the indicated time points p.i., 5 mice were euthanized and blood was harvested by cardiac puncture for measurements of various soluble mediators including VEGFA (A), MMP-9 (B), IL-6 (C) and TNF-α (D). Legend: DENV2-infected mice born to DENV1 immune mothers (black bar); DENV2-infected mice born to dengue naive mothers (white bar); uninfected age-matched mice born to dengue naïve mothers (hatched bar). ND, not detectable. *, p<0.05.

The current paradigm of DEN-associated vascular leakage involves a number of soluble players including inflammatory cytokines and chemokines, as well as non-inflammatory soluble mediators, whereby elevated serum levels of these mediators have been correlated with disease severity [41], [42]. Key mediators including VEGF, MMP-9, IL-6 and TNF-α were measured and compared between both groups of DENV2-infected mice born to either DENV1 immune or naive mothers. A significantly higher level of VEGF was measured at Day 5 p.i. in DENV2-infected mice born to DENV1 immune mothers compared to uninfected controls (Fig. 5A). However, this level was not significantly different from that measured in DENV2-infected mice born to dengue naïve mothers. No significant differences in the levels of MMP-9 were observed between both DENV2 infected groups and compared to the uninfected controls (Fig. 5B). In contrast, the levels of IL-6 and TNF-α were significantly higher at day 3 and 5 p.i. in mice born to DENV1 immune mothers compared to those measured in mice born to naïve mothers. Furthermore, when comparing both DENV2-infected groups at moribund stage, mice born to DENV1 immune mothers displayed significantly higher levels of IL-6 and TNF-α than mice born to naïve mothers (Fig. 5C&D). Of note, the cytokine levels measured in age-matched uninfected mice born to naïve or DENV1 immune mothers were comparable (data not shown).

These results thus suggest that a greater inflammation reaction is triggered in DENV2-infected mice born to DENV1 immune mothers, as evidenced by higher levels of IL-6 and TNF-α.

### Role of TNF-α in increased vascular leakage in DENV2-infected mice born to DENV1 immune mothers

**Figure 6:**
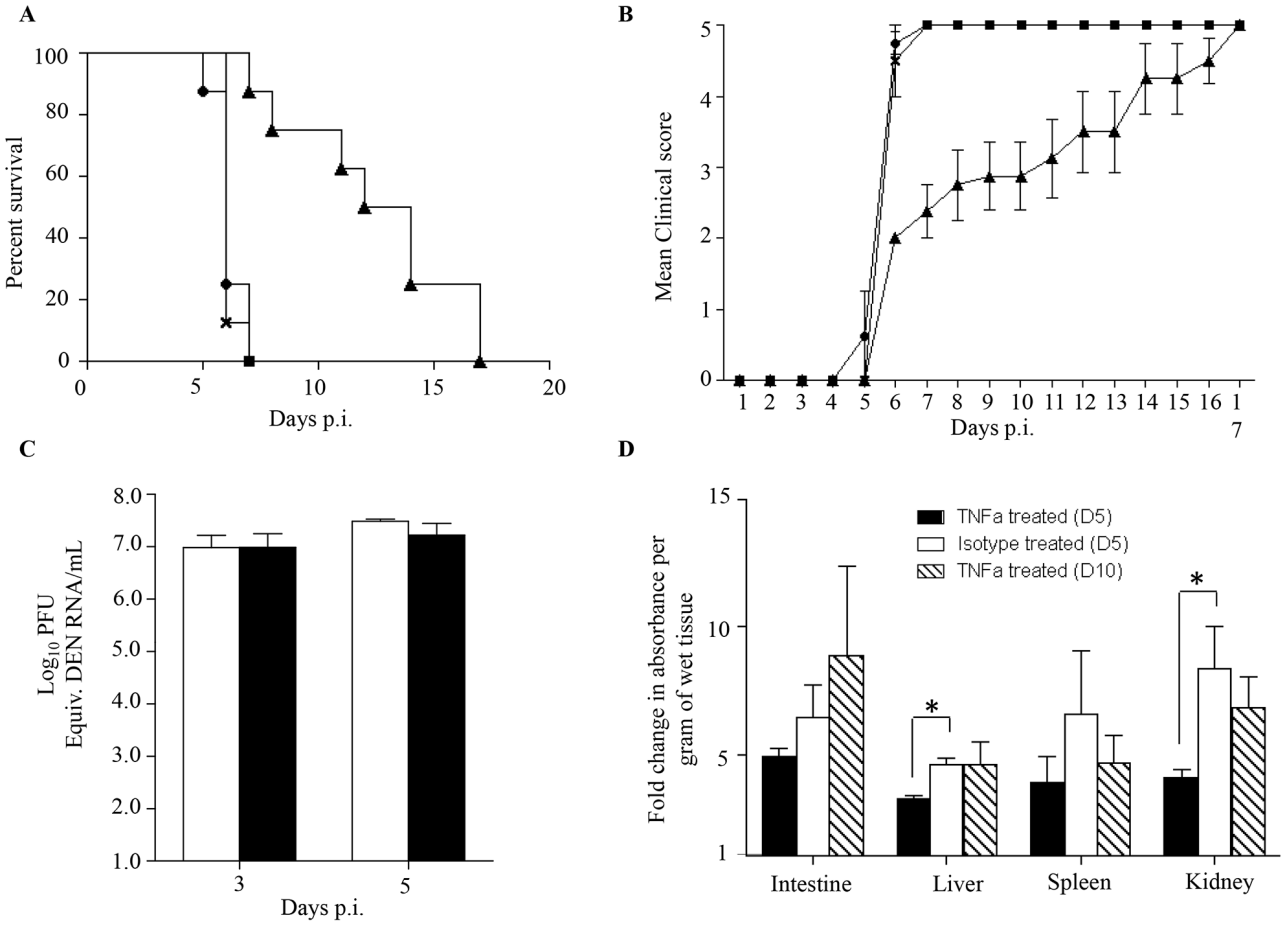
TNF-α neutralization in DENV2-infected mice born to DENV1 immune or naïve mothers. 5-weeks old AG129 mice born to DENV1 immune mothers were sc. infected with 10^3^ PFU of D2Y98P-PP1. At day 1 and 2 p.i., 100 µg of anti-TNFα neutralizing antibodies (solid triangle) or 100 µg of isotype antibody control (cross) were injected intraperitoneally. A control group of mice not injected was also included (solid circle). A) Survival rate (n = 10 mice per group). B) Clinical score (n = 10) 0: Healthy; 1: Ruffled Fur; 2: Hunched back; 3: Severe Diarrhea; 4: Lethargic; 5: Moribund. C) Viremia. At day 3 and 5 p.i., 5 mice per group per time point were euthanized and bled by cardiac puncture. The virus loads were determined by real-time PCR. Legend: DENV2-infected mice treated with isotype control (white bar); DENV2-infected mice treated with anti-TNFα antibodies (black bar). D) Vascular leakage. At day 5 (black and white bars), or day 10 (hatched bar) p.i., mice (n = 5) treated with anti-TNFα (black and hatched bars) or isotype (white bar) antibodies were euthanized and Evan's blue extravasation assay was performed. See legend of Fig. 1D. *, p<0.05.

Elevated levels of TNF-α in severe dengue patients have been measured [43]–[45]. Furthermore, the role of TNF-α in dengue disease severity has been demonstrated whereby the administration of anti-TNFα blocking antibodies delayed the fatal outcome of DENV-infected AG129 mice [25], [37], [46]. To confirm the role of TNF-α in our mouse model of enhanced dengue disease severity, DENV2-infected mice born to DENV1 immune mothers were administered with anti-TNFα blocking antibodies at day 1 and 2 p.i. Survival rates were monitored and indicated that anti-TNFα antibody treatment delayed the animals' death significantly compared to untreated animals and compared to mice treated with an isotype antibody control, with survival means of 13, 6 and 6 days respectively (Fig. 6A). Appearance of clinical symptoms was also delayed in the anti-TNFα antibody treated mice (Fig. 6B).

To further investigate the effects of anti-TNFα antibody treatment, viremia was determined at day 3 and 5 p.i. No significant difference in viremia was observed at day 3 and 5 p.i. between the anti-TNFα and isotype control-treated groups (Fig. 6C).

The involvement of TNF-α in dengue-associated vascular leakage has been shown *in vitro* [47], [48] but has yet to be directly demonstrated in an *in vivo* model of severe dengue. Here, vascular permeability was thus measured in both the anti-TNFα and isotype control treated groups at day 5 and day 10 p.i. The results indicated that the extent of vascular leakage was significantly lower at day 5 p.i. in the liver and kidneys from the anti-TNFα treated mice compared to the isotype control group (Fig. 6D). However, at day 10 pi. vascular leakage in the anti-TNFα treated mice was comparable to that measured in the isotype control group at day 5 p.i (Fig. 6D).

Altogether these results demonstrate that the anti-TNFα antibody treatment given at day 1 and 2 p.i. resulted in transient and partial control of vascular leakage which correlated with delayed death of the animals. This set of data provides a direct experimental evidence of a role for TNF-α in increased vascular permeability in an *in vivo* model of severe dengue.

### Role of maternally acquired DENV1 antibodies in shifting disease severity from asymptomatic to lethal DENV2 infection

**Figure 7:**
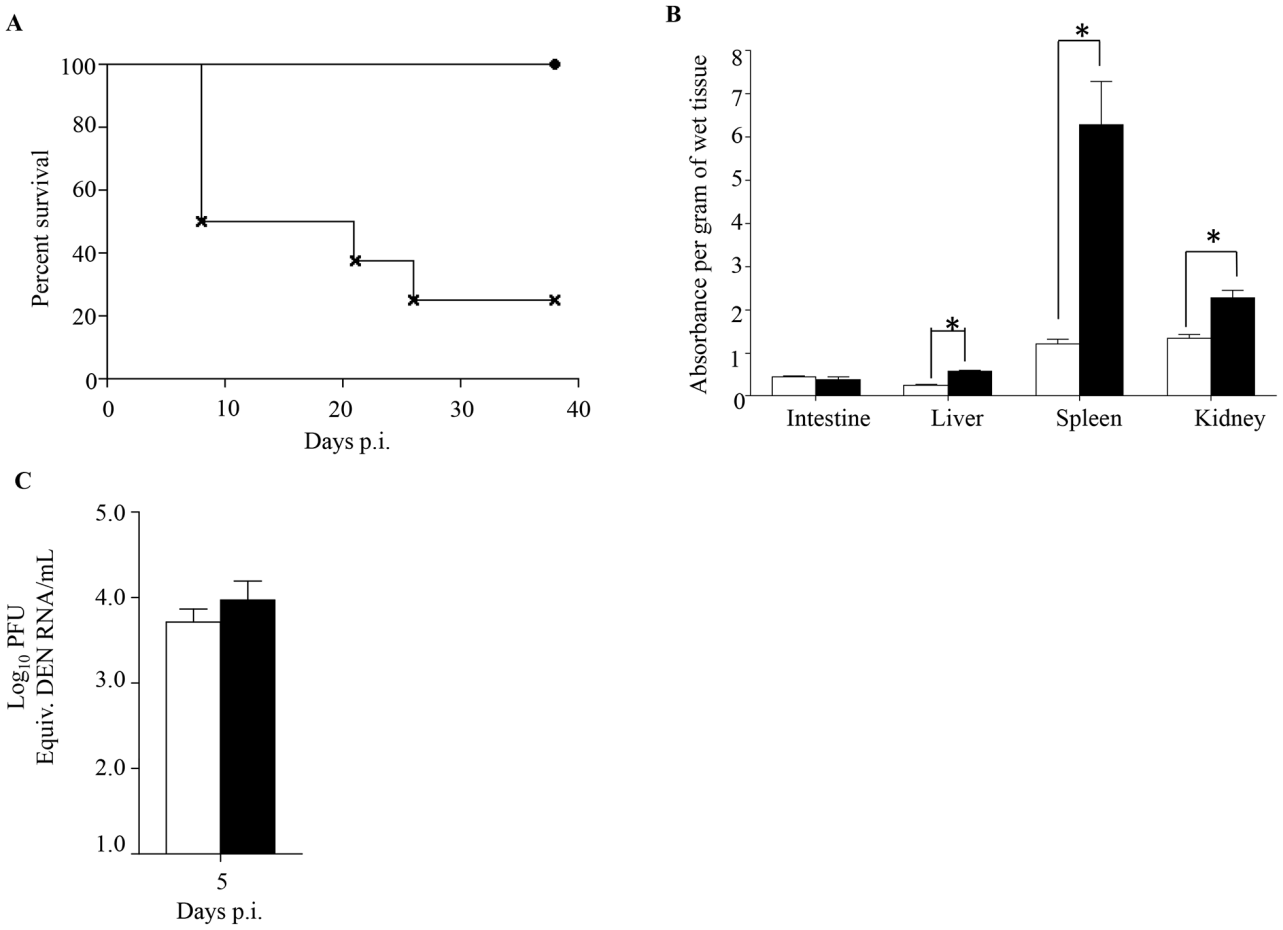
Survival rate, vascular leakage and viremia in mice born to either DENV1 immune or dengue naïve mothers, and infected with MT5 DENV2. 5-weeks old AG129 mice born to either DENV1 immune or dengue naïve mothers were sc. infected with 10^3^ PFU of MT5 DENV2 strain. A) Survival Rate (n = 10 mice per group). Legend: MT5-infected mice born to dengue naïve (black circle); MT5-infected mice born to DENV1 immune mothers (cross). B) Vascular leakage. Moribund MT5-infected mice born to DENV1 immune mothers (black bar) were euthanized and Evan's blue extravasation assay was performed. Vascular leakage was compared to MT5-infected mice born to dengue naïve mothers (white bar) (n = 5 mice per group). C) Viremia was measured by real time PCR at Day 5 p.i. in MT5-infected mice born to DENV1 immune (black bar) or naïve (white bar) mothers (n = 5 mice per group). *, p<0.05.

Since primary infection with any of the 4 DENV serotypes primarily results in asymptomatic disease, we tested the ability of maternally acquired DENV1 antibodies to shift the disease outcome from asymptomatic to severe disease upon DENV2 infection. To do so, 5-week old mice born to DENV1 immune or naïve mothers were infected with a DENV2 mutant virus strain (namely MT5) that was derived from the lethal DENV2 D2Y98P-PP1 strain through a single amino acid substitution in NS4B protein [33]. This amino acid substitution resulted in lower viral replication efficacy which translated into asymptomatic transient viremia upon primary infection in dengue naïve adult AG129 mice ([33] and Fig. 7A). However, infection with 10^3^ PFU of MT5 virus of 5-week old mice born to DENV1 immune mothers resulted here in lethal outcome for more than 70% of the animals (Fig. 7A). These animals displayed hunched back, progressive lethargy and diarrhea, but no paralysis. In addition, significant vascular leakage was measured in the liver, spleen and kidneys from these animals at moribund stage (Fig. 7B), supporting a correlation between disease severity and increased vascular permeability. However, surprisingly viremia measured at day 5 p.i. in MT5-infected mice born to DENV1 immune mothers was not statistically different from the viremia measured in MT5-infected mice born to dengue naïve mothers (Fig. 7C). We postulate that the lack of statistical difference may be due to the heterogeneity in both disease outcome and time of disease manifestations observed with the MT5-infected mice born to DENV1 immune mothers.

Thus, this set of data demonstrates that maternally acquired heterotypic DENV antibodies are able to shift dengue disease outcome from asymptomatic to severe, thereby recapitulating epidemiological observations.

### Serum from mice born to DENV1 immune mothers cross-reacts with DENV2 and enhances DENV2 infection *in vitro*

**Figure 8:**
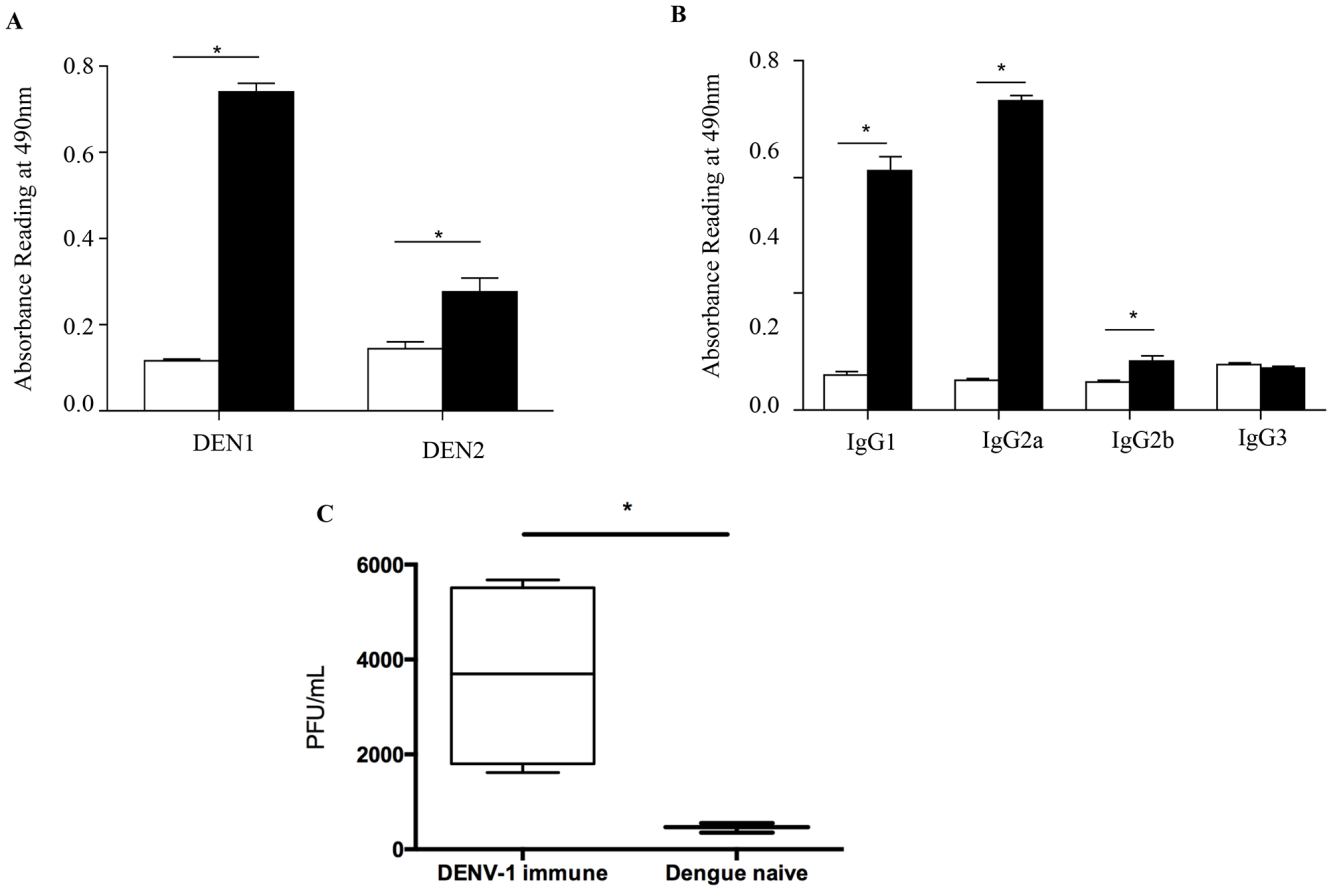
*In vitro* ELISA and ADE assays with the serum from mice born to DENV1 immune mothers. A) Total IgG antibodies against DENV1 or DENV2 were determined by ELISA in the serum from 5-week old mice born to DENV1 immune (black bar) or dengue naïve (white bar) mothers (n = 5 mice per group). B) Anti-DENV1 IgG antibody subclasses were analyzed by ELISA. Legend: serum from mice born to naïve mothers (white bar); serum from mice born to DENV1 immune mothers (black bar). C) Antibody-Dependent Enhancement (ADE) assay. Sera diluted 1/20 from 5-week old mice born to DENV1 immune or dengue naïve mothers (n = 5 mice per group) were mixed with DENV2 virus prior to infection of THP-1 macrophages. At 3 days p.i., the number of infectious particles produced in the supernatant was determined by plaque assay in BHK-21 cells. *, p<0.05.

To further characterize the nature and properties of the maternal DENV1 specific antibodies circulating in 5-week old mice born to DENV1 immune mothers, ELISA was carried out using UV-inactivated DENV1 or DENV2 particles as coating antigens. Expectedly, elevated absorbance readings were measured when probing for the presence of anti-DENV1 total IgG antibodies in the serum from 5-week old mice born to DENV1 immune mothers (Fig. 8A). In addition, serum from these mice cross-reacted significantly with DENV2 (Fig. 8A). Isotyping of the anti-DENV1 IgG antibodies revealed majority of IgG1 and IgG2a circulating in the serum of mice born to DENV1 immune mothers (Fig. 8B).

To further confirm the role in disease severity enhancement of the maternal antibodies circulating in mice born to DENV1 immune mothers at the time of DENV2 infection, an *in vitro* ADE assay was carried out as reported before [38]. Neat serum samples from 5-week old mice born to DENV1 immune or naive mothers were mixed with DENV2 prior to infection of Fcγ-R bearing macrophages THP-1. At 3 days p.i., the number of infectious particles produced in the supernatant was determined by plaque assay. Serum samples from mice born to DENV1 immune mothers clearly enhanced DENV2 production compared to the serum samples from mice born to dengue naïve mothers (Fig. 8C). These data thus confirmed the enhancing properties of the serum from 5-week old mice born to DENV1 immune mothers.

### Characterization of the age windows of protection versus disease enhancement in mice born to DENV1 immune mothers

**Figure 9:**
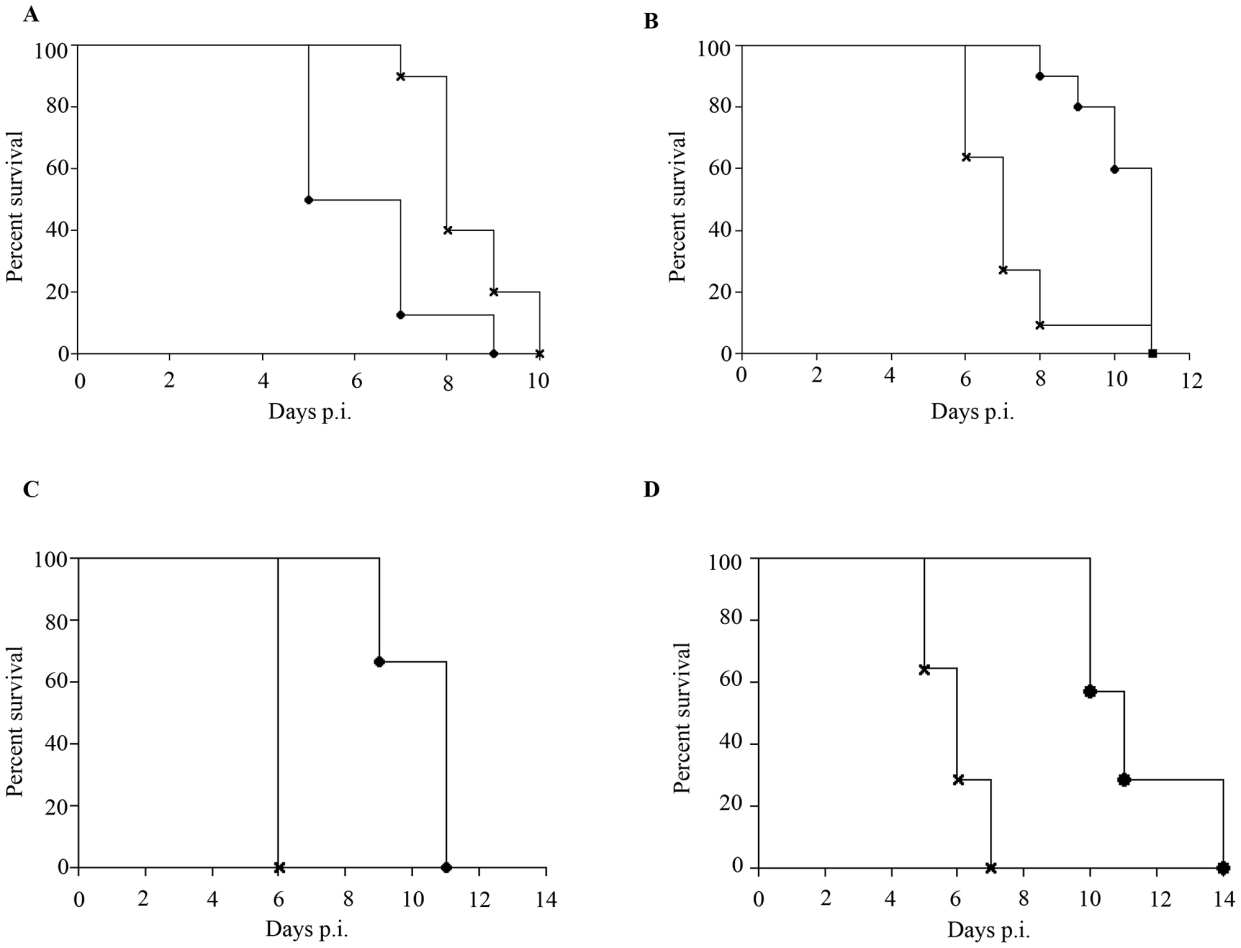
Disease protection versus disease enhancement age windows. AG129 mice born to DENV1 immune (cross) or dengue naïve (black circle) mothers were infected with 10^3^ PFU of D2Y98P-PP1 at 2 weeks (A), 3 weeks (B), 5 weeks (C) or 8 weeks (D) of age. Survival rates were monitored and compared. (n = 8–10 mice per group).

**Figure 10:**
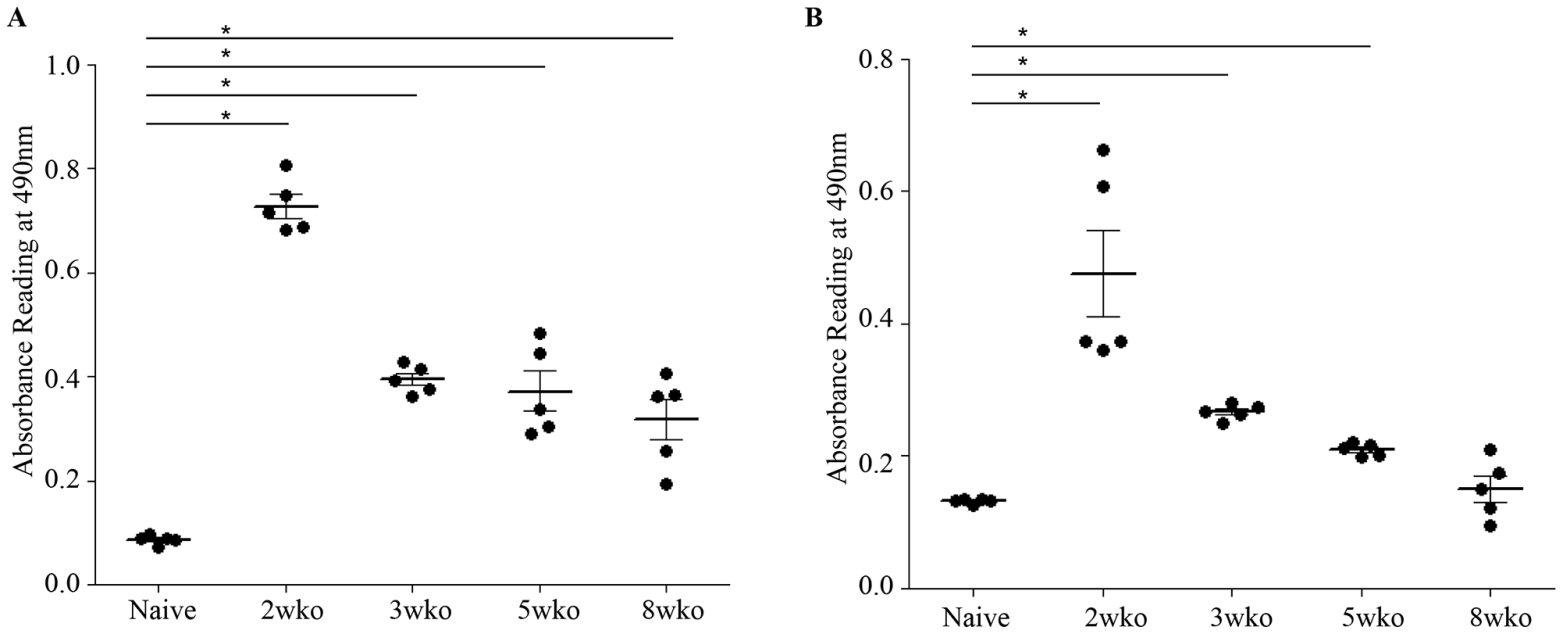
Age-dependent anti-DENV1 and anti-DENV2 IgG antibody responses in mice born to DENV1 immune mothers. The anti-DENV1 (A) and anti-DENV2 (B) total IgG antibody responses were monitored by ELISA in AG129 mice born to DENV1 immune mothers at 2 weeks, 3 weeks, 5 weeks or 8 weeks of age as indicated. A group of 5-week old mice from dengue naïve mothers was also used as control. N = 5 mice per group. *, p<0.05.

Epidemiological studies reported that specifically 5–9 months old infants born to dengue immune mothers are at risk of developing enhanced dengue disease severity upon primary DENV infection [12]. To explain this age window of disease enhancement, it was proposed that the level of maternally acquired DENV specific antibodies initially provides the baby with a pan-serotype protection against DENV infection. However, as the level of maternal antibodies wanes over time, the maternal antibodies no longer neutralize but instead enhance infection with a heterologous or even homologous DENV serotype. In an attempt to recapitulate in our mouse model this age-dependent disease protection versus enhancement status, DENV2 challenge was performed in mice born to DENV1 immune mothers at 2 weeks, 3 weeks, 5 weeks or 8 weeks of age. The survival rates showed that 2-week old mice born to DENV1 immune mothers were significantly protected against DENV2 challenge compared to age-matched animals born to dengue naïve dams (Fig. 9 panel A). In contrast, 3-week, 5-week and 8-week old mice born to DENV1 immune mothers died significantly earlier than age-matched animals born to dengue naïve mothers (Fig. 9 panels B–D). Furthermore, the level of maternal DENV1 IgG antibodies measured in 2-week old mice was significantly higher than the level measured in 3-week, 5-week and 8-week old animals (Fig. 10 panel A). Of note, comparable levels of anti-DENV1 IgG antibodies were measured in 3, 5 and 8 week-old animal groups which suggests that the half-life of these antibodies is longer than the average 2 weeks half-life previously reported for rodent IgG antibodies [49]. In contrast, cross-reactivity with DENV2 of the serum from mice born to DENV1 immune mothers clearly declined with age, suggesting that cross-reactive anti-DENV1 antibody species may have a shorter half-life than non cross-reactive species (Fig. 10 panel B). Finally, the neutralizing activity against DENV2 determined by PRNT showed that the serum from 2-week old mice displayed a strong neutralizing activity against DENV2 (PRNT_50_ above 50) whereas the PRNT_50_ measured with the sera from 3-, 5- and 8-week old mice were below 20 (Table 1). Therefore, both the ELISA and PRNT data correlate well with the disease outcomes observed upon DENV2 challenge in mice born to DENV1 immune mothers, and support the epidemiological observations in DENV-infected infants born to DENV immune mothers.

**Table 1:** Neutralizing activity against DENV2 of the serum from 2–8 week-old mice born to DENV1 immune mothers.

Age	2-week old	3-week old	5-week old	8-week old
PRNT_50_	54.64	17.29	13.85	18.53

Serial dilution (2-fold) of the serum from 2, 3, 5 and 8 week-old mice born to DENV1 immune mothers were mixed with DENV2 prior to infection of BHK-21 cells. The number of plaques obtained was visually scored and compared to the number of plaques obtained with the virus alone (positive control). The plaque reduction neutralizing titer (PRNT_50_) was determined by nonlinear regression as the serum dilution factor for which 50% reduction in the number of plaques was obtained compared to the positive control.

## Discussion

Advancement in understanding DEN pathogenesis has been largely hampered by the lack of a suitable animal model. Humans and mosquitoes are so far the only known natural hosts for DENV. Upon DENV infection, non-human primates develop viremia and produce neutralizing antibody responses, but they do not display overt clinical signs of disease [11]. Moreover ethic and economic considerations have greatly limited the use of non-human primates as animal model to study DEN. Immune competent mice are not susceptible to DENV infection [11]. Indeed, while DENV was shown to interfere and block IFN signalling in human cells [50], [51], it fails to do so in murine counterparts [52]. Accordingly, immune-compromised mice which lack type I and II IFN signalling pathways (AG129) were found permissive to infection with most of DENV lab strains and clinical isolates whereby transient viremia could be detected [11], [53]. However, clinical manifestations in these mice are DENV strain-dependent and range from none (majority of the DENV strains) to severe vascular leakage accompanied by thrombocytopenia [11]. Recently, our group has reported the subcutaneous infection of adult AG129 mice with a non mouse-adapted DENV2 strain, namely D2Y98P-PP1 [36]. In this model, the virus disseminates systemically and replicates transiently in the blood and a variety of organs. Severe vascular leakage develops over time which eventually leads to non-paralytic death of the infected animals after the virus has been cleared from the blood circulation [36]. However, the lack of functional type I & II IFN responses represents an important weakness for this dengue mouse model since dengue patients are generally immune competent. Data generated in this mouse model must therefore be interpreted with caution, and may not accurately reflect the situation in patients. A recent work has indicated the susceptibility of IFNAR (Type I IFN) KO mice to infection with a DENV2 strain [54]. It would be very interesting to test whether these mice would display maternal antibody-mediated enhancement of disease severity as seen in AG129 mice.

Leveraging on our model of primary DENV2 infection in AG129 mice, we report here a model of ADE that resembles the situation observed in humans, where infants born to DENV immune mothers have greater risk to develop severe dengue disease. This is the first report of an *in vivo* ADE model that demonstrates the role of maternally acquired heterotypic DENV antibodies in disease severity. Previous *in vivo* models of ADE have relied on the passive transfer of DENV specific antibodies (immune sera or monoclonal antibodies) followed by heterotypic DENV infection, a somewhat indirect and artificial approach [23]–[25]. Here, DENV2 infection of 5-week old AG129 mice born to DENV1 immune mothers displayed enhancement of dengue disease severity as evidenced by earlier time of death, increased virus loads in the circulation and in the spleen, and increased vascular leakage compared to DENV2-infected mice born to dengue naïve mothers. Similar disease pattern and kinetic was observed upon passive transfer of DENV1 immune serum to DENV2-infected mice, thus strongly supporting the role of DENV1 specific antibodies in disease severity enhancement. Furthermore, mimicking even more closely the situation in humans, using an attenuated DENV2 strain (MT5) derived from the lethal D2Y98P-PP1 strain, we showed that mice born to DENV1 immune mothers developed lethal dengue accompanied by vascular leakage upon MT5 infection whereas MT5-infected mice born to dengue naïve mothers did not display any clinical manifestation.

Importantly, we were able to observe in this mouse model the age-dependent susceptibility to severe disease as described in humans [12], whereby 2-week old mice born to DENV1 immune mothers were significantly protected upon DENV2 challenge, whereas 3, 5 or 8 week-old mice displayed disease severity enhancement. These disease outcomes correlated well with the levels of maternal DENV1 specific IgG antibodies and their neutralizing activity against DENV2 measured in the serum from the mice born to DENV1 immune mothers. These observations suggest that unlike humans, mice seem to have a rather large susceptibility window for disease enhancement due to the sustained presence of maternally acquired DENV1 antibodies at enhancing concentrations. This may indicate that the half-life of these maternal DENV1 IgG antibodies may be greater than the average half-life of 2 weeks previously suggested for rodents IgG antibodies [49]. In humans, a byphasic decay pattern has been reported for maternal dengue IgG antibodies with a half-life of 24–29 days between birth and 3 months, and 44–150 days after 3 months [55]. It is thus possible that in mice, maternally acquired DENV1 specific IgG antibodies may display a similar biphasic decay pattern.

Higher viremia was measured at day 5 p.i. in DENV2-infected mice born to DENV1 immune mothers compared to DENV2-infected mice born to dengue naïve mothers. This observation supports the findings from several epidemiological studies reporting that higher viremia was measured in DHF cases arising from secondary heterologous DENV infection [12], [39], [40]. Our model here supports experimentally that high viremia correlates with disease severity, although this dogma has recently been challenged by recent epidemiological studies where no correlation could be established between viremia levels and disease severity [32], [56].

Using *in vitro* ELISA and ADE assays, we clearly showed that the serum from mice born to DENV1 immune mothers cross-reacted with DENV2 particles and eventually enhanced DENV2 replication in Fc-R bearing cells. Thus, our ADE mouse model seems to support the ADE hypothesis. After an initial brief period of cross-reactive neutralization, possibly due to the high maternal antibody titer that co-ligates the inhibitory FcγRIIB [38], the virus-antibodies complexes facilitated infection of myeloid cells, such as monocytes, through activating Fc-R to result in increased virus replication and output [16]–[18]. In order for an antibody to be neutralizing and therefore protective in the presence of Fc-R-mediated entry, it must be of high enough affinity to be able to neutralize epitopes at the surface of the virus and must be in sufficient concentration [57]. Failure to fulfill either of these requirements results in enhancement of the disease. This idea can explain why antibodies induced by one DENV serotype, although protective against that serotype [58], may increase the risk of severe disease upon infection with a heterologous DENV serotype due to enhanced infection of FcR-bearing cells by sub-neutralized viral particles [59]. It was proposed that due to differences in the surface antigens between DENV serotypes, only some of the antibodies raised against one will react with another, and among these cross-reactive antibodies, some may have a reduced affinity for the second serotype. Therefore, during a heterotypic DENV infection, the threshold for neutralization is less likely to be reached and, consequently, ADE-mediated severe disease is more likely to occur.

At the intracellular level, it was proposed that FcR-mediated entry of DENV allows the virus to escape the type I and II IFN intracellular signaling pathways, thereby resulting in greater virus replication [18], [60]. However, higher viremia was seen in this ADE model that uses AG129 mice which are deficient for both type I and II IFN pathways. This suggests therefore that the enhanced viral output observed in this ADE mouse model results from the viral escape of type I and II IFN-independent antiviral mechanisms.

Elevated serum levels of an ever increasing list of soluble mediators (such as TNFα, IL-6, C5a, VEGF, MMP-9 and others) have been measured in DHF/DSS patients and represent hallmarks of severe dengue. The current paradigm of DEN-associated vascular leakage involves the combination of a plethora of soluble factors (including pro-inflammatory cytokines/chemokines and other non-inflammatory mediators) that act in concert and lead to the transient alteration of the vascular permeability [41], [42]. Surprisingly, only IL-6 and TNF-α levels were found higher in DENV2-infected mice born to DENV1 immune mothers compared to DENV2-infected mice born to dengue naïve mothers, despite the significantly higher vascular leakage measured in the former group. However, a more exhaustive list of mediators should be measured and this work is currently in progress in our laboratory.

Using neutralizing antibodies, we demonstrated the role of TNF-α in this ADE mouse model whereby anti-TNFα antibody treatment resulted in transient and partial control of vascular leakage in some organs which correlated with delayed death of the animals, while it had no impact on the viremia. The correlation between elevated systemic levels of TNF-α and dengue disease severity has been reported in a number of *in vivo* mouse model of severe dengue [24], [25], [35], [36], [37], [46], [54], [61], among which four of them have used anti-TNFα antibody treatment to demonstrate a reduced mortality rate [25], [37], [46], [61]. However, these studies failed to report an impact of the anti-TNFα treatment on vascular leakage specifically. Thus our work provides the *in vivo* direct evidence of a role for TNF-α in DENV-induced vascular leakage in an ADE model.

In addition to the cytokine storm theory (which has the weakness to be observed in other acute infections without leading to increased vascular permeability), other mechanisms have been proposed to play a role in DENV-induced vascular leakage. Among possible mechanisms, circulation of DENV specific antibodies capable of cross-reacting with host proteins has been reported and referred to as DENV-induced autoimmunity [62]. Specifically, Anti-NS1, anti-E, anti-prM and anti-C antibodies were shown to cross-react with endothelial cells, platelets, and some of the molecules involved in the coagulation and fibrinolysis pathways [62]. Involvement of these antibodies in enhanced vascular permeability observed in our ADE mouse model are currently being investigated in our laboratory.

In conclusion, this study is a long-overdue experimental demonstration of the ADE hypothesis proposed by Halstead as early as 1970 [12], [13]. This mouse model reproduces the natural route of maternal antibodies transfer from DENV1 immune mothers to their offspring and how they impact on dengue disease severity upon DENV2 infection. Despite the caveat of the absence of the type I and II IFN pathways, our mouse model offers a unique opportunity to study a number of aspects that are specifically involved in maternal antibody-mediated enhancement of dengue disease severity. For example, the dynamics of the IgG placental transfer, the role and importance of DENV specific antibodies acquired through breast milk, or the half-life of the transferred maternal antibodies can be studied in this model. Also very importantly, the impact of DENV vaccination of mothers on the offspring can be addressed. Furthermore, several studies have reported a higher neutralizing DENV antibody titer in cord blood versus maternal serum, suggesting a preferential movement across the placenta of some DENV antibody sub-species with greater avidity for DENV [63], [64]. This aspect can be specifically addressed in our model and the identification of the IgG antibody sub-species that preferentially cross the placenta might reveal very useful.

In addition we believe that that this mouse model offers a novel platform for therapeutic testing in the context of ADE that can be more relevant than the previously described ADE models which rely on the administration of enhancing concentrations of DENV specific antibodies prior to DENV infection. Indeed, while the latter rely on the administration of antibody doses that may be totally outside the range of concentrations that would be actually present in naturally infected dengue individuals, the maternal antibody-mediated ADE model avoids such bias. This aspect is of particular importance when testing the possible interference of pre-existing DENV specific antibodies on the protective efficacy of some therapeutic candidates in particular monoclonal antibodies that would compete with pre-existing circulating antibodies for binding to the virus particles.

## Supporting Information

Figure S1 **Viremia profile in DENV1 infected AG129 mice.** Female adult AG129 mice were sc. infected with 10^5^ PFU of a DENV1. At the indicated time points post-infection, 5 mice were euthanized and blood was collected. The virus titers in the sera were determined by plaque assay in BHK-21 cells.
(PPTX)
