# Supplementary figures and images for: First Experimental In Vivo Model of Enhanced Dengue Disease Severity through Maternally Acquired Heterotypic Dengue Antibodies
Source: PLoS Pathog. 2014 Apr 3;10(4):e1004031. doi: 10.1371/journal.ppat.1004031 (PMC3974839; doi:10.1371/journal.ppat.1004031)

## Slide 1
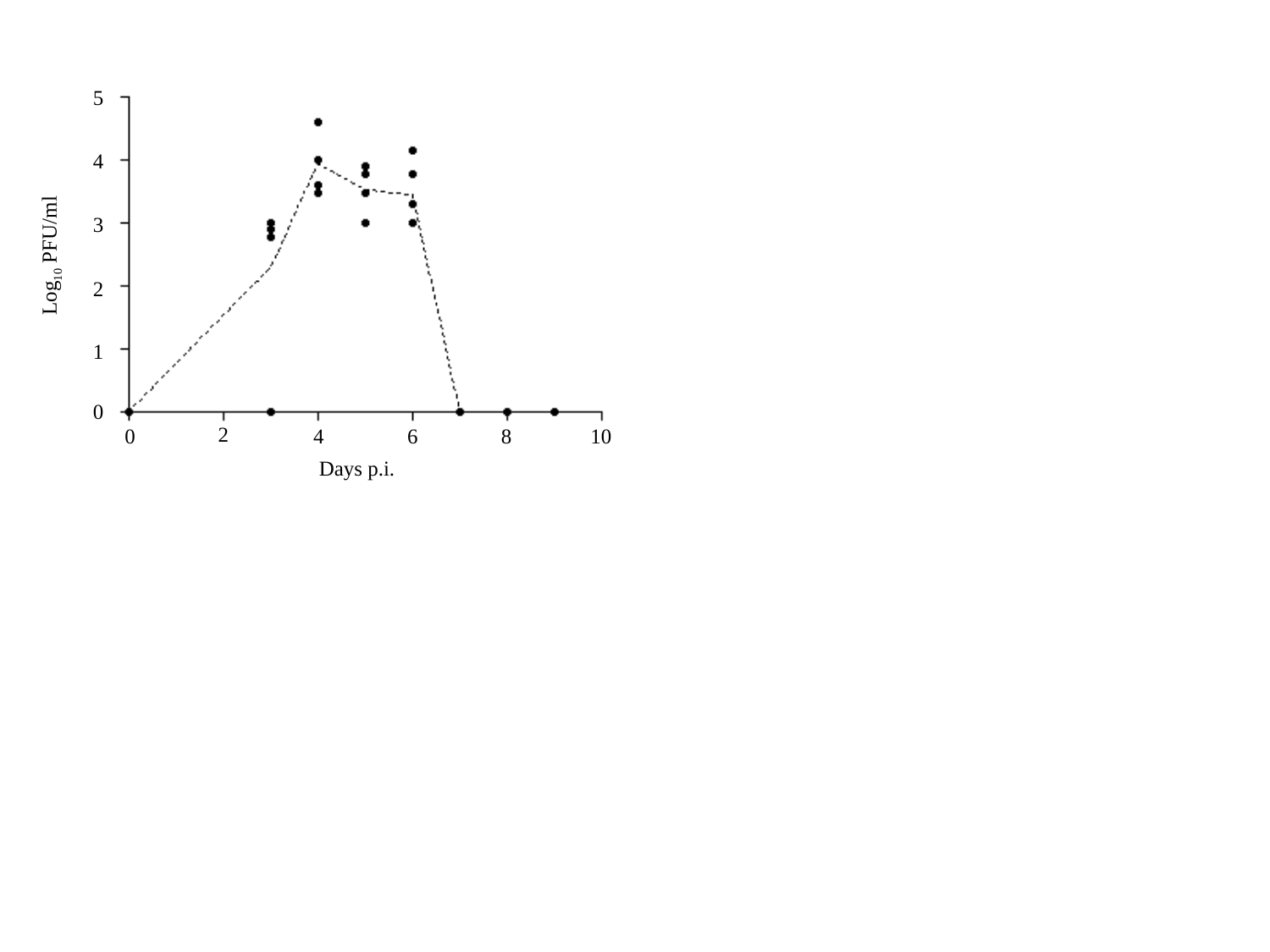

5
4
Log10 PFU/ml
3
2
1
0
2
0
4
6
8
10
Days p.i.

Supplement: Figure S1 — Viremia profile in DENV1 infected AG129 mice. Female adult AG129 mice were sc. infected with 105 PFU of a DENV1. At the indicated time points post-infection, 5 mice were euthanized and blood was collected. The virus titers in the sera were determined by plaque assay in BHK-21 cells. (PPTX) [file ppat.1004031.s001.pptx]
